# Comprehensive Phenolic Profiling and Antioxidant Evaluation of Calyx, Rind, and Edible Tissues From Non‐Astringent Persimmons (*Diospyros kaki*)

**DOI:** 10.1002/cbdv.71492

**Published:** 2026-07-27

**Authors:** Huiwen Wang, Muhammad Sajid Arshad, Ayesha Siddiqa, Malik Adil Nawaz, Hafiz A. R. Suleria

**Affiliations:** ^1^ School of Agriculture Faculty of Science The University of Melbourne, Food and Ecosystem Science Parkville Victoria Australia; ^2^ School of Agriculture and Food Sustainability The University of Queensland St Lucia Queensland Australia

**Keywords:** antioxidants, Fuyu, Jiro, bioactive compounds, LC‐ESI‐QTOF‐MS/MS, persimmon

## Abstract

Persimmon (*Diospyros kaki* L.) processing generates substantial quantitiesof underutilized by‐products despite their richness in bioactive compounds. This study evaluated the phenolic content, antioxidant potential, and phytochemical composition of four tissues (calyx, rind, pulp, inner pulp) from two non‐astringent cultivars, namely, Fuyu and Jiro. Phenolic estimation (TPC, TFC, and TTC) and seven antioxidant assays (DPPH, ABTS^+^, ·OH‐RSA, FRAP, RPA, TAC, and FICA) revealed strong tissue‐specific variation. Calyx tissues displayed the highest phenolic concentrations and antioxidant activities, with Fuyu calyx showing the greatest TPC (38.98 mg GAE/g DW) and TTC (46.32 mg CE/g). Edible tissues exhibited substantially lower values, consistent with tannin insolubilization during ripening. Pearson's correlations showed strong associations between TPC/TFC and all antioxidant assays (*r* = 0.985−0.999), except FICA, which demonstrated weak negative correlations, highlighting method limitations for metal‐chelating evaluation in persimmon matrices. LC‐ESI‐QTOF‐MS/MS profiling identified 95 phenolic compounds across samples, including hydroxybenzoic acids, hydroxycinnamic acids, flavonoids, lignans, and stilbenes, with calyx tissues containing the greatest diversity. HPLC‐DAD quantification confirmed gallic acid, catechin hydrate, and sinapic acid as key compounds, detected in Fuyu calyx. Overall, persimmon calyx represents a potent source of antioxidant phenolics with strong potential for valorization in functional food and nutraceutical applications.

## Graphical abstract

Tissue‐specific phenolic profiling of non‐astringent persimmons (Fuyu and Jiro) reveals substantial differences in phytochemical composition and antioxidant potential. Persimmon calyx exhibits the highest concentrations of bioactive compounds, highlighting its potential as a valuable by‐product for functional food and nutraceutical applications.

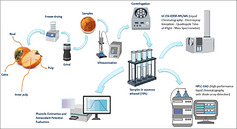

## 1 Introduction

Persimmon (*Diospyros kaki* L.) is an economically and nutritionally valuable fruit native to East Asia and now cultivated globally (Jain et al. 2023) [1]. Beyond its sweetness and carotenoid‐rich profile, persimmon is increasingly recognized for its diverse phytochemicals including polyphenols, flavonoids, carotenoids, and tannins which contribute to antioxidant, anti‐inflammatory, antidiabetic, and hypolipidemic activities [2]. Although most studies have focused on the edible pulp, emerging evidence indicates that non‐edible tissues such as the peel, leaves, calyx, and seeds also contain substantial quantities of bioactive compounds and hold promise for functional ingredient development [3].

Cultivar type plays a significant role in determining the chemical composition of persimmon tissues. Astringent cultivars (e.g., ‘Hachiya’) accumulate high levels of soluble tannins and proanthocyanidins, resulting in strong antioxidant potential but requiring postharvest de‐astringency. In contrast, non‐astringent cultivars such as ‘Fuyu’ and ‘Jiro’ naturally induce tannin in‐solubilization during maturation, producing sweeter fruit with reduced astringency [4]. These physiological differences influence both phenolic distribution and antioxidant function, highlighting the importance of cultivar‐specific characterization.

Simultaneously, global persimmon production faces sustainability challenges, as large quantities of calyx, peel, and other tissues are discarded during harvesting and processing despite their considerable phytochemical richness [5]. In the context of growing interest in circular bio‐economy frameworks, these underutilized by‐products constitute promising candidates for value‐added upcycling, contingent upon comprehensive characterization of their compositional and functional attributes.

However, most previous studies have focused on persimmon pulp and peel, whereas non‐edible tissues such as the calyx remain poorly investigated. Furthermore, comparative information on the tissue‐specific distribution of phenolic compounds and antioxidant activities in commercially important non‐astringent cultivars such as ‘Fuyu’ and ‘Jiro’ is limited. Therefore, a comprehensive comparison of different persimmon tissues is needed to better understand their phytochemical composition and valorization potential.

In this study, two commercially important non‐astringent persimmon cultivars, Fuyu’ and ‘Jiro’, were selected to evaluate tissue‐specific variations in phenolic composition and antioxidant potential. Calyx, rind, outer pulp, and inner pulp tissues were analyzed using phenolic content assays (TPC, TFC, and TTC), antioxidant assays, LC‐ESI‐QTOF‐MS/MS profiling, and HPLC‐DAD quantification. The aim was to identify phenolic‐rich tissues and assess their potential as sources of value‐added functional ingredients.

## 2 Material and Methods

### 2.1 Fruit Samples, Chemicals, and Reagents

Fresh fruits of *Diospyros kaki* L. cultivars ‘Fuyu’ and ‘Jiro’ were purchased from Woolworths, Melbourne, Victoria, Australia. The cultivar identity was verified based on supplier information and fruit morphological characteristics. Fuyu fruits were supplied by Sunland Fresh Fruit (Cobram, Victoria, Australia) through The N&A Fruit Distributors, Sydney Markets, Australia, whereas Jiro fruits were supplied by Vitor Marketing (Riverland, South Australia, Australia) and grown by Kangara Farm, Paringa, South Australia, Australia. Only fruits with uniform appearance, size, and commercial maturity were selected for analysis to minimize biological variation among samples. Moreover, analytical‐grade reagents, standards, and chemicals used for phenolic and antioxidant analyses were purchased from Sigma‐Aldrich (Castle Hill, NSW, Australia) including gallic acid, L‐ascorbic acid, vanillin, hexahydrate aluminum chloride, Folin–Ciocalteu's phenol reagent, sodium phosphate, iron(III) chloride hexahydrate (FeCl_3_.6H_2_O), hydrated sodium acetate, hydrochloric acid, anhydrous sodium carbonate, ammonum molybdate, quercetin, catechin, 2,2′‐diphenyl‐1‐picrylhydrazyl (DPPH), 2,4,6‐tripyridyl‐s‐triazine (TPTZ), 2,2′‐azinobis‐(3‐ethylbenzothiazoline‐6‐sulfonic acid) (ABTS), iron(II) sulfate heptahydrate, hydrogen peroxide, 3‐hydroxybenzoic acid, sodium phosphate monobasic monohydrate, potassium ferricyanide, trichloroacetic acid, iron(III) chloride, iron(II) chloride, 3‐(2‐pyridyl)‐5,6‐diphenyl‐1,2,4‐triazine‐*p,p*′‐disulfonic acid monosodium salt (ferrozine), ethylenediaminetetraacetic acid (EDTA), and solid sodium hydroxide (NaOH). Organic reagents including methanol, ethanol, acetonitrile, formic acid, and were purchased from Thermo Fisher Scientific Inc. (Scoresby, VIC, Australia).

### 2.2 Sample Preparation

Fresh persimmon fruits were manually washed and separated into four tissues: calyx, rind (peel), outer pulp, and inner pulp. Four tissues from each cultivar (Fuyu and Jiro) were collected, resulting in a total of eight sample groups, designated as Fuyu calyx (CFC), Fuyu rind (CFR), Fuyu pulp (CFP), Fuyu inner pulp (CFV), Jiro calyx (MJC), Jiro rind (MJR), Jiro pulp (MJP), and Jiro inner pulp (MJV). The samples were stored at −80°C for 72 h prior to freeze‐drying using a Zirbus VaCo 5 freeze dryer (ZIRBUS Technology, Germany). Freeze‐dried samples were ground into powder and stored in sealed containers at −20°C until further analysis.

### 2.3 Extraction of Polyphenolic Compounds

For extraction of phenolic rich extracts, samples were weighed at 3±0.01 g followed by mixing with 20 mL of ethanol. After that, ultrasonication was performed in the ultrasonic bath and sonicated at 20 kHz at 55% amplitude for 3 min (Qsonica Q55, 55 W, USA). Samples were then placed in shaking incubation for 24 h under room temperature (∼22°C). Once completion, the samples were subjected for centrifuge at Hettich Refrigerated Centrifuge (ROTINA380R, Tuttlingen, Baden‐Württemberg, Germany) at 4,651 rpm for 10 min at 23°C. Subsequently, these supernatants were collected and stored in sterile containers at 4°C for stock. The extracts were further filtered through 0.45 µm syringe filter (Thermo Fisher Scientific Inc., Waltham, MA, USA) for HPLC and LC‐MS analysis.

### 2.4 Characterization of Phenolic Compounds in Persimmons

#### 2.4.1 Estimation of Phenolic Content

Polyphenol estimation in persimmon samples extract were performed employing in vitro assay (TPC, TFC, and TTC) using 96‐well plate method in triplicates. The results were provided by a Multiskan Go microplate photometer (Thermo Fisher Scientific, Waltham, MA, USA).

#### 2.4.2 Total Phenolic Content (TPC) Assay

The method for determining the total phenolic content (TPC) was optimized from Suleria, Barrow [6]. Briefly, in a 96‐well microplate, 25 µL of the sample and 25 µL of Folin's reagent were mixed together, and then 200 µL of distilled water was added. Following incubation for 5 min at room temperature (∼22°C), 25 µL of 10% (w/w) sodium carbonate was added, and the mixture was further incubated for an additional 60 min at 25°C. Finally, absorbance was measured at 765 nm. Gallic acid solutions (0–200 µg/mL in ethanol) were used to generate a standard curve, and the results were reported as mg of gallic acid equivalents (GAE) per gram of dry sample.

#### 2.4.3 Total Flavonoids Content (TFC) Assay

Aluminum chloride colorimetric method established by Suleria, Barrow [6] was used to determine the total flavonoid content (TFC). 96‐well microplate was filled with 80 µL of the extracted sample, 80 µL of a 2% aluminum chloride solution, and 120 µL of a 50 g/L sodium acetate solution followed by 2.5 h of incubation at room temperature (∼22°C). A microplate reader was used to measure the absorbance at 440 nm. Quercetin solutions (0–50 µg/mL in methanol) were used to generate a standard curve, and the results were reported as mg of quercetin equivalents (QE) per gram of dry sample.

#### 2.4.4 Total Tannin Content (TTC)

Total content of tannin (TTC) was determined by the use of the vanillin‐sulfuric acid method by Suleria, Barrow [6]. Generally, 150 µL of 4% vanillin solution and 25 µL of 32% sulfuric acid were added into 25 µL of the sample in a 96‐well plate. Absorbance was measured at 500 nm following a 15 min incubation at 25°C. A standard curve was generated using catechin solutions (0–1000 µg/mL in methanol), and the results were reported as mg catechin equivalents (CE) per gram of dry sample.

### 2.5 Antioxidant Potential Evaluation of Persimmon‐Derived Phenolic Compounds

The antioxidant properties of the persimmon phenolic extracts were evaluated through seven different assays, including DPPH, FRAP, ABTS, RPA, ^·^OH‐RSA, FICA, and TAC. DPPH and ABTS assays were included to evaluate radical scavenging capacity through hydrogen atom and single electron transfer mechanisms. FRAP and RPA were used to assess reducing potential and electron‐donating ability of phenolic constituents. ·OH‐RSA was included to measure hydroxyl radical scavenging activity against one of the most reactive oxygen species. FICA evaluated ferrous ion chelation capacity, an important indirect antioxidant mechanism by limiting metal‐catalyzed oxidative reactions. Finally, TAC provided an integrative assessment of the overall antioxidant potential of the extracts. Three duplicates of each test were conducted in 96‐well plates using previously published procedures and parameters with some modifications. A Multiskan Go microplate spectrophotometer (Thermo Fisher Scientific, Waltham, MA, U.S.A.) was used to measure the sample absorbance.

#### 2.5.1 DPPH Free‐Radical Scavenging Activity Assay

The free‐radical scavenging activity of persimmon samples was measured using in vitro assay [7]. Generally, 96‐well plates were filled with 275 µL of DPPH methanol solution (0.1 M) and 25 µL of sample solution. The triplicate mixture was then set in the dark room to incubate for 30 min and measured at 517 nm. trolox solutions with concentrations ranging from 0 to 100 µg/mL were used to generate the standard curve, and the results were reported as mg trolox equivalents (TE) per gram of dry sample.

#### 2.5.2 Ferric Reducing Antioxidant Power (FRAP) Assay

By applying the previously published techniques by Suleria, Barrow [6], the FRAP procedure was used to determine the ferric reducing activity of persimmon samples, which estimates the capacity of persimmon samples to convert the Fe^3+^‐TPTZ complex to the Fe^2+^‐TPTZ complex. To prepare the FRAP reagent, 10 mM TPTZ, 20 mM ferric chloride, and 300 mM sodium acetate buffer (pH = 3.6) were mixed in a 1:1:10 (v/v/v) ratio. A 96‐well plate was filled with 20 µL of the persimmon sample solution and 280 µL of FRAP reagent. The mixture was incubated in dark for 10 min at room temperature. The absorbance was measured at 593 nm. Trolox solutions with concentrations ranging from 0 to 100 µg/mL were used to generate the standard curve, and the results were reported as mg trolox equivalents per gram of dry sample.

#### 2.5.3 ABTS Radical Scavenging Assay

With some modifications, the previously published techniques of Severo, Tiecher [8] were used to evaluate the radical scavenging activity of persimmon samples. Generally, 7 mM ABTS solution and 140 mM potassium persulfate solution was fully mixed, and then the mixture was incubated for 16 h in a dark environment. Following confirmation of around 0.75 absorbance at 734 nm, 290 µL of ABTS solution and 10 µL of persimmon extract solution were mixed in a 96‐well plate and incubated for 6 min at room temperature. The absorbance was measured at 734 nm. Ascorbic acid solutions with concentrations ranging from 0 to 150 µg/mL were used to generate the standard curve, and the results were reported as mg ascorbic acid equivalents (AAE) per gram of dry sample.

#### 2.5.4 Reducing Power Assay (RPA)

The RPA test was used to evaluate the reducing power of persimmon samples, a modified version of the method according to Ferreira, Baptista [9] was used. Generally, a 96‐well plate was filled with 25 µL of 0.2 M phosphate buffer (pH 6.6), 10 µL of sample extract solution, and 25 µL of 1% (w/v) K_3_[Fe (CN)_6_]. The plate was then allowed to incubate at room temperature for 20 min. Following the addition of 8.5 µL of 0.1% (w/v) FeCl_3_ and 85 µL of water, the mixture was incubated for a further 15 min at room temperature before 25 µL of 10% TCA solution was added to prevent the reaction. The absorbance was measured at 750 nm. Trolox solutions with concentrations ranging from 0 to 500 µg/mL were used to generate the standard curve, and the results were reported as mg trolox equivalents per gram of dry sample.

#### 2.5.5 Hydroxyl Radical Scavenging Activity (OH‐RSA)

The hydroxyl radical scavenging ability of persimmon samples was assessed using the ^·^OH‐RSA assay [7]. Generally, in a 96‐well plate, 50 µL of 6 mM H_2_O_2_, 50 µL of 6 mM FeSO_4_·7H_2_O, and 50 µL of the sample solution were initially added. After the addition of 50 µL of 6‐methylbenzoic acid, the absorbance of the sample was finally measured at 510 nm. Ascorbic acid solutions with concentrations ranging from 0 to 400 µg/mL were used to generate the standard curve, and the results were reported as mg ascorbic acid equivalents per gram of dry sample.

#### 2.5.6 Ferrous Ion Chelating Activity (FICA)

The FICA test was used to evaluate the metal chelation activity of persimmon using the previously published method of Ali, Bashmil [10]. Generally, a 96‐well plate was filled with 50 µL of 5 mM ferrozine water solution (1:6, v/v), 50 µL of 2 mM ferrous chloride water solution (1:15, v/v), 85 µL of water, and 15 µL of the sample solution. The plate was then incubated at room temperature for 10 min. The absorbance was measured at 562 nm. Ethylenediaminetetraacetic acid (EDTA) solutions with concentrations ranging from 0 to 50 µg/mL were used to generate the standard curve, and the results were reported as mg ascorbic acid equivalents per gram of dry sample.

### 2.6 Phenolic Profiling via LC‐QTOF‐MS/MS

To further precipitate the potential existence of proteins and polysaccharides, extracted samples were processed. Generally, 80% of methanol was added to each sample in a 1:4 (v/v) ratio. They were then centrifuged for 10 min at 12000 × g after being incubated at 4°C overnight. A 0.45 µm filter was used to eliminate any remaining precipitation from the collected supernatant. Pure methanol in 1:20 was used to further dilute the samples.

An Agilent 1200 Infinity HPLC system in conjunction with an Agilent 6520 Q‐TOF MS fitted with an ESI source was used to profile polyphenols. Phenolic separation was performed using a Synergi Hydro‐RP column (250 × 4.6 mm, 4 µm) with a C18 guard column. Then, a 1 µL injection volume was used. 0.1% formic acid was used in water (A) and acetonitrile (B) for elution, with a flow rate of 0.8 mL/min. The gradient was 1%–2% B (0–4 min), 2%–5% B (4–10 min), 5%–45% B (10–50 min), 45%–98% B (50–52 min), 98% B (52–54 min), and 2% B (54–56 min). DAD at 210–290 nm and Q‐TOF in both ESI+ and ESI− modes (*m/z* 50–1300) were used for detection. With capillary and nozzle voltages of 3.5 kV and 500 V, respectively, nitrogen was utilized as the drying gas (5 L/min, 300°C, 45 pressure).

### 2.7 HPLC‐DAD Quantitative Analysis

Fifteen phenolic standards were selected, including epigallocatechin gallate, 3,4‐dihydroxycinnamic acid, 3‐hydroxy‐4‐methoxycinnamic acid, epicatechin, 4‐hydroxybenzoic acid, gallic acid, syringic acid, sinapic acid, quercetin, oleanolic acid, ellagic acid, p‐coumaric acid, catechin hydrate, *trans*‐ferulic acid, protocatechuic acid, naringin, procyanidin A2, caffeic acid, *trans*‐cinnamic acid, pyrogallol, and chlorogenic acid.

Depending on their solubility at 1 mg/mL, standards were made in ethanol, DMSO, or water. An Agilent 1200 Series system (Agilent Technologies, CA) with a diode array detector (DAD) was used to conduct the HPLC study. A Synergi Hydro‐Reverse Phase 80 Å, LC column (250×4.6 mm, 4 µm, Phenomenex, Torrance, CA) equipped with a C18 ODS guard column (Phenomenex, Torrance, CA) with an internal diameter of 4.0×2.0 mm was used to accomplish standards separation. With a steady flow rate of 0.5 mL/min, the mobile phase was prepared by mixing up of solvents A, which was made of 0.1% formic acid in water, and B, which was made of 0.1% formic acid acetonitrile. The injection volume was 10 µL for both standard and sample runs. The gradient performed as the following: 2% B for 0–2 min; 2%–45% B for 2–17 min; 45%–60% B for 17–32 min; 60%–70% B for 32–32.5 min; 98% B for 35.5–36.5 min; 98% B for 36.5–38.5 min; 98%–2% B for 38.5–39.0 min; and 2% B for 39–42 min.

The calculation was using the following equation:

$$\begin{array}{ccc} \mathrm{Conc}.\;\mathrm{of}\;\mathrm{phenolic}\;\mathrm{content}= & & \frac{\mathrm{Peak}\;\mathrm{area}\;\mathrm{of}\;\mathrm{sample}\;\mathrm{extract}}{\mathrm{Peak}\;\mathrm{area}\;\mathrm{of}\;\mathrm{phenolic}\;\mathrm{standard}}\\ & & \times \mathrm{Conc}.\;\mathrm{of}\;\mathrm{phenolic}\;\mathrm{standard} \end{array}$$

### 2.8 Statistical Analysis

All experiments were performed in triplicate (*n* = 3), and results are expressed as mean ± standard deviation (SD). Data were examined for normality and homogeneity of variance before statistical analysis. One‐way analysis of variance (ANOVA) was used to evaluate differences among samples, followed by Tukey's honestly significant difference (HSD) post hoc test. Statistical significance was accepted at *p* < 0.05. GraphPad Prism (Version XX, GraphPad Software, USA) was used for statistical comparisons, while Pearson's correlation analysis was performed using Minitab software.

## 3 Results and Discussion

### 3.1 Estimation of Phenolic Profile

#### 3.1.1 Total Phenolic Content (TPC)

The total phenolic content (TPC) of persimmon tissues is presented in Table 1, revealing significant differences among the four tissue types and between the two non‐astringent cultivars, Fuyu and Jiro. In both cultivars, the calyx exhibited markedly higher TPC than all other tissues, with Fuyu calyx showing 38.98 ± 0.96 mg GAE/g DW and Jiro calyx 29.38 ± 0.93 mg GAE/g DW. This pronounced enrichment aligns with the biological role of the calyx as a protective, phenolic‐rich barrier exposed to environmental stressors, consistent with reports that peripheral and structural tissues typically accumulate higher concentrations of phenolics as part of plant defense responses [11].

**TABLE 1:** Antioxidant properties in two non‐astringent persimmon cultivars from four different parts.

Persimmon variety	Parts	Phenolic profile			Antioxidant potential						
		TPC (mg GAE/g)	TFC (mg QE/g)	TTC (mg CE/g)	DPPH (mg AAE/g)	FRAP (mg TE/g)	ABTS+ (mg AAE/g)	RPA (mg TE/g)	^·^OH‐RSA (mg AAE/g)	FICA (mg EDTA/g)	TAC (mg TE/g)
Fuyu	Calyx	38.98 ± 0.96^a^	0.56 ± 0.01^b^	46.32 ± 2.48^a^	129.79 ± 8.73^a^	92.35 ± 6.79^a^	59.35 ± 2.16^a^	46.02 ± 0.57^a^	189.84 ± 4.06^a^	0.15 ± 0.01^d^	28.39 ± 1.99^a^
	Rind	2.3 ± 0.03^c^,^d^	0.17 ± 0.01^d^	—	7.66 ± 0.33^c^	4.17 ± 0.30^b^	7.13 ± 0.16^d^	4.19 ± 0.25^d^,^e^	58.13 ± 0.04^b^	0.37 ± 0.02^b^	3.37 ± 0.32^b^
	Pulp	1.96 ± 0.01^c^,^d^	0.02 ± 0.01^f^	—	5.18 ± 0.46^c^	4.54 ± 0.03^b^	4.74 ± 0.24^d^,^e^	3.36 ± 0.15^f^	55.38 ± 0.41^b^,^c^	0.27 ± 0.01^c^	4.52 ± 0.19^b^
	Inner pulp	1.02 ± 0.04^d^	0.10 ± 0.01^e^	—	1.82 ± 0.05^c^	0.60 ± 0.05^b^	2.96 ± 0.02^e^	1.46 ± 0.10^g^	54.39 ± 0.38^b^,^c^	0.41 ± 0.00^a^	2.53 ± 0.07^b^
Jiro	Calyx	29.38 ± 0.93^b^	0.66 ± 0.01^a^	38.73 ± 0.96^b^	117.06 ± 4.96^b^	89.69 ± 3.45^a^	53.86 ± 2.03^b^	42.40 ± 0.04^b^	186.72 ± 3.89^a^	0.16 ± 0.01^d^	27.25 ± 2.55^a^
	Rind	2.95 ± 0.02^c^	0.28 ± 0.01^c^	—	9.63 ± 0.69^c^	5.32 ± 0.02^b^	6.10 ± 0.19^d^	4.61 ± 0.02^d^	50.88 ± 0.17^c^,^d^	0.27 ± 0.01^c^	3.11 ± 0.25^b^
	Pulp	2.58 ± 0.02^c^	0.04 ± 0.01^f^	—	7.32 ± 0.50^c^	6.81 ± 0.61^b^	6.95 ± 0.31^d^	5.77 ± 0.19^c^	46.87 ± 1.74^d^	0.16 ± 0.01^d^	5.16 ± 0.05^b^
	Inner pulp	2.03 ± 0.02^c^,^d^	0.10 ± 0.01^e^	—	6.41 ± 0.26^c^	3.59 ± 0.12^b^	10.71 ± 0.08^c^	3.59 ± 0.14^e^,^f^	49.74 ± 1.74^c^,^d^	0.28 ± 0.02^c^	2.99 ± 0.10^b^

*Note*: *Results are expressed in mean ± standard deviation. All results are expressed in dry weight. Values with different letters (^a–g^) within the same column are significantly different (*p* < 0.05). TPC values are expressed as GAE (gallic acid equivalent) in mg/g; TFC values are expressed as QE (quercetin equivalent) in mg/g; TTC values are expressed as (CE) catechin equivalent in mg/g; FRAP, RPA, and TAC values are expressed as TE (trolox equivalent) in mg/g; DPPH, ABTS^+^ values are expressed as AAE (ascorbic acid equivalent); FICA values are expressed as mg EDTA (ethylenediaminetetraacetic acid equivalent)/g; ^·^OH^−^ values are expressed as AAE (ascorbic acid equivalent) in mg/g.

Abbreviations: TPC, total phenolic content; TFC, total flavonoid content; DPPH, 2,2‐diphenyl‐1‐picrylhydrazyl radical scavenging activity; FRAP, ferric reducing antioxidant power; ABTS+, 2,2'‐azino‐*bis*(3‐ethylbenzothiazoline‐6‐sulfonic acid) radical scavenging activity; RPA, reducing power activity; ^·^OH, hydroxyl radical scavenging activity; FICA, ferric ions chelating activity; TAC, total antioxidant capacity.

In contrast, edible tissues contained significantly lower phenolic levels. The inner pulp exhibited the lowest TPC (Fuyu: 1.02 ± 0.04 mg GAE/g; Jiro: 2.03 ± 0.02 mg GAE/g). These reductions reflect ripening‐associated biochemical changes, including oxidative degradation of phenolics, dilution by increasing moisture and sugars, and the polymerization of tannins into insoluble cell‐wall‐bound complexes, which markedly decrease solvent‐extractable phenolics [2, 12, 13].

Rind tissues showed intermediate TPC values, with Fuyu at 2.30 ± 0.03 mg GAE/g and Jiro at 2.95 ± 0.02 mg GAE/g. This observation is consistent with the characteristic enrichment of phenolics in epidermal tissues exposed to light, which enhances phenylpropanoid‐derived biosynthesis, especially of chlorogenic acids and flavonols [14].

Comparison between cultivars showed that Jiro generally contained higher TPC in edible tissues, whereas Fuyu calyx had higher phenolic content than Jiro calyx. These variations likely arise from genotypic differences in phenolic biosynthesis, metabolism, and the rate of tannin insolubilization [2].

When compared with previous work, the TPC values reported here for Fuyu calyx are lower than the 81.81 mg GAE/g reported by Jang, Jo [15]. Similar discrepancies were observed for peel and pulp. Despite numerical differences, the tissue distribution pattern (calyx > rind > pulp) remained consistent. The lower values in the present study may be attributed to differences in extraction solvents (70% ethanol vs. absolute ethanol), fruit maturity (fully ripened fruits contain fewer soluble phenolics), and tissue moisture content, all of which influence extraction efficiency. Due to limited studies on the Jiro cultivar, comparative data for calyx and rind remain scarce. One study reported lower TPC in fresh Jiro pulp (1.68 mg GAE/g), potentially due to differing extraction solvents (acidified methanol) and sample forms (fresh vs. freeze‐dried).

#### 3.1.2 Total Flavonoid Content (TFC)

Total flavonoid content (TFC), determined using the aluminum chloride colorimetric method, showed a distribution pattern similar to TPC but with markedly lower absolute values across all tissues (Table 1). As with phenolics overall, the calyx exhibited the highest flavonoid concentrations, reaching 0.56 ± 0.01 mg QE/g in Fuyu and 0.66 ± 0.01 mg QE/g in Jiro. This enrichment is consistent with the functional role of outer floral structures as major sites of flavonoid accumulation, where these compounds contribute to UV protection, pigmentation, and pathogen defense [11, 14].

Flavonoid content in edible tissues was extremely low. Pulp contained only 0.02 ± 0.01 mg QE/g in Fuyu and 0.04 ± 0.01 mg QE/g in Jiro, while inner pulp exhibited slightly higher values (0.10 ± 0.01 mg QE/g in both cultivars). These concentrations are negligible relative to outer tissues, reflecting the reduced requirement for photoprotective compounds in internal fruit structures and the decline of extractable flavonoids during ripening. The relatively low TFC values observed in the present study may also be influenced by extraction conditions and the analytical specificity of the aluminum chloride colorimetric assay. This method primarily detects flavonoids capable of forming complexes with aluminum ions and may underestimate certain flavonoid subclasses. Furthermore, non‐astringent persimmon cultivars undergo substantial biochemical changes during ripening, which can reduce the concentration of extractable flavonoids in edible tissues. Similar reductions in flavonoid content during fruit maturation have been reported in previous studies on persimmon and other fruit matrices.

Rind tissues showed intermediate but notable TFC values (Fuyu: 0.17 ± 0.01 mg QE/g; Jiro: 0.28 ± 0.01 mg QE/g), consistent with the common localization of flavonoids in epidermal layers exposed to light and oxidative stress [11]. Across all tissues, Jiro displayed higher flavonoid content than Fuyu, suggesting cultivar‐specific differences in the regulation of the shikimate and proanthocyanidin biosynthetic pathways, which influence both total flavonoid accumulation and PA composition [16].

Compared with previously published values, the TFC in persimmon rind reported here is comparable to the unusually high rind‐to‐pulp ratio of flavonoids observed by [17]. However, differences in expression units (e.g., mg kaempferol/g), extraction conditions, and the limited number of studies examining TFC in persimmon tissues restrict direct quantitative comparison with earlier work.

#### 3.1.3 Total Tannin Content (TTC)

The tissue‐specific distribution of polyphenols was further supported by the total tannin content (TTC). Tannins were quantifiable only in the calyx of both cultivars, with no detectable levels in the rind, pulp, or inner pulp. Fuyu calyx contained 46.32 ± 2.48 mg CE/g, while Jiro calyx showed a slightly lower value of 38.73 ± 0.96 mg CE/g. These findings align with previous work indicating that tannins become largely undetectable in persimmon fruit tissues after ripening [18, 19]. Only one earlier study quantified TTC in persimmon calyx, reporting 30–40 mg CE/g in astringent cultivars at maturation [18], comparable to the present results. However, due to the scarcity of TTC studies in non‐astringent cultivars, broader comparisons remain limited.

The absence of measurable tannins in the edible tissues of mature fruits is attributed to well‐established physiological and biochemical processes occurring during ripening [2, 12]. In immature persimmons, soluble condensed tannins accumulate in vacuoles, where they contribute to astringency through interactions with salivary proteins [20]. As the fruit ripens, these tannins undergo a series of transformations polymerization, oxidative crosslinking, and complexation with cell‐wall polysaccharides such as pectins and hemicelluloses—forming high‐molecular‐weight, water‐insoluble aggregates [21, 22]. In parallel, tannin‐protein complexes precipitate through colloidal aggregation [23], further reducing their extractability.

Non‐astringent cultivars exhibit an additional genetically driven mechanism: natural de‐astringency initiated early in maturation. This process involves acetaldehyde‐mediated in solubilization of soluble tannins, rendering them undetectable by colorimetric or solvent‐based extraction despite their structural persistence within the tissue matrix [22].

The high TTC observed in calyx tissue suggests that this floral structure does not undergo the same degree of tannin in‐solubilization as the fruit flesh, allowing residual extractable condensed tannins to remain. These tannins likely contribute to the strong antioxidant activity observed in calyx extracts. The higher TTC in Fuyu relative to Jiro calyx may reflect tissue‐specific differences in tannin retention, biosynthesis, or degradation kinetics, as previously reported in persimmon floral tissues [13].

### 3.2 Estimation of Antioxidant Potential

The antioxidant capacity of persimmon tissues was evaluated using seven complementary in vitro assays encompassing three major antioxidant mechanisms. Free radical scavenging activity was assessed using DPPH, ABTS^+^, and hydroxyl radical (·OH‐RSA) assays; non‐radical redox potential was measured through the FRAP, RPA, and TAC assays; and metal‐chelating capacity was determined using the FICA assay. Together, these assays provide a comprehensive assessment of hydrogen‐donating ability, electron‐transfer capacity, and ferrous‐ion chelation across calyx, rind, pulp, and inner pulp tissues. The complete results for all antioxidant assays are summarized in Table 1.

#### 3.2.1 Free Radical Scavenging Ability (DPPH, ABTS^+^, and ·OH)

The DPPH assay, which measures the capacity of antioxidants to donate hydrogen atoms or electrons to neutralize the stable DPPH radical (Shi et al. 2025) [24] revealed pronounced differences in scavenging activity among persimmon tissues. Calyx tissues exhibited the strongest activity, with Fuyu and Jiro showing 129.79  and 117.06 mg AAE/g, respectively. This result is consistent with the high phenolic and tannin content of calyx tissues, which strongly contributes to radical‐quenching capacity through electron‐transfer and hydrogen‐donation mechanisms [25].

In contrast, edible tissues demonstrated substantially lower DPPH activity. Inner pulp showed only 1.82 mg AAE/g in Fuyu and 6.41 mg AAE/g in Jiro, while pulp exhibited 5.18 and 7.32 mg AAE/g, respectively. The slightly higher values in Fuyu reflect its greater retention of extractable phenolics, consistent with the TPC trends observed earlier. Rind samples displayed intermediate activity (Fuyu: 7.66 mg AAE/g; Jiro: 9.63 mg AAE/g), reflecting moderate phenolic accumulation in epidermal tissues exposed to light and environmental stress, which stimulate flavonoid biosynthesis [11, 14].

Across both cultivars, the pattern of antioxidant activity followed the order calyx > rind > pulp > inner pulp, which aligns with previous findings for non‐astringent persimmons [15, 26]. However, direct numerical comparison with earlier studies is limited because DPPH values were often expressed as IC_50_ rather than antioxidant equivalent units.

The ABTS^+^ assay is widely used to evaluate free radical scavenging via electron or hydrogen atom transfer to the ABTS^+^ radical cation, which forms upon oxidation and is responsive in both aqueous and lipid media [27]. In this study, calyx tissues again showed the highest ABTS^+^ values (Fuyu: 59.35  mg AAE/g; Jiro: 53.86  mg AAE/g), significantly exceeding those of rind, pulp, and inner pulp. Owing to the assay's compatibility with both hydrophilic and amphiphilic antioxidants, ABTS^+^ is particularly sensitive to compounds such as phenolic acids, ascorbic acid, and water‐soluble flavonoids [27]. Interestingly, Jiro exhibited markedly higher ABTS^+^ activity in the edible tissues, with the inner pulp (10.71 mg AAE/g) showing more than threefold the activity of Fuyu (2.96 mg AAE/g), whereas Fuyu showed slightly greater activity in the rind (7.13 mg AAE/g) compared with Jiro (6.10 mg AAE/g). This pattern suggests that Jiro may retain a higher proportion of hydrophilic antioxidants (e.g., free phenolic acids such as caffeic acid) during ripening, reflecting cultivar‐dependent metabolic differences and supported by our LC‐ESI‐QTOF‐MS/MS profiling [2, 28]. Although direct comparison with prior studies is limited due to different units (e.g., IC_50_ or µmol TE/100 g), the tissue‐level trend (calyx > rind > pulp ≥ inner pulp) aligns with previous reports in non‐astringent persimmons [15, 26]. The relatively lower ABTS^+^ values in Fuyu edible tissues, despite higher overall TPC in some cases, may also reflect assay sensitivity and radical specificity differences [27].

The hydroxyl radical scavenging assay, based on a Fenton reaction system (Fe^2^^+^/H_2_O_2_), further revealed the capacity of persimmon tissues to neutralize highly reactive ·OH species [29]. Both Fuyu and Jiro calyx samples exhibited exceptionally strong ·OH scavenging (189.84 and 186.72 mg AAE/g DW, respectively), in line with their elevated polyphenolic and condensed tannin contents. Among edible tissues, both cultivars showed moderate ·OH‐RSA, with Fuyu generally outperforming Jiro in rind (58.13 vs. 50.88 mg AAE/g DW), pulp (55.38 vs. 46.87 mg AAE/g DW), and inner pulp (54.39 vs. 49.74 mg AAE/g DW). The persistence of ·OH‐RSA despite lower TPC in edible tissues indicates that activity depends not only on total phenolic levels but also on the presence and accessibility of specific structural motifs (e.g., catechol/galloyl groups in flavonoids and phenolic acids) [29, 30]. Similar ·OH‐RSA values between pulp and inner pulp suggest a relatively homogeneous distribution of hydrophilic antioxidants within mature non‐astringent fruit tissues. Because Fenton‐based ·OH assays can also be influenced by metal chelation (thereby reducing ·OH generation), the overall rank generally follows ABTS^+^ and DPPH (calyx > rind > pulp > inner pulp), with minor deviations potentially arising from matrix constituents (e.g., benzoic acid derivatives) that consume reagents or modulate reaction kinetics [29].

#### 3.2.2 Metal Chelating Ability (FICA)

Unlike DPPH or FRAP, which involve electron‐transfer mechanisms, the FICA assay is based on metal complexation, primarily involving the catechol groups on the *B*‐ring of flavonoids and the 3‐hydroxyl groups of phenolic acids [31]. Ferric ion chelating activity reflects the ability of a compound to bind Fe^2^^+^ and form ferrous complexes, thereby preventing metal‐catalyzed Fenton reactions that generate highly reactive hydroxyl radicals [31].

In this study, Fuyu inner pulp exhibited the highest FICA of 0.41 mg EDTA/g, followed by rind at 0.37 mg EDTA/g, pulp at 0.27 mg EDTA/g, and calyx at 0.15 mg EDTA/g. A similar trend was observed in Jiro, with FICA values of 0.28 mg EDTA/g, 0.27 mg EDTA/g, and 0.16 mg EDTA/g in the inner pulp, rind, and calyx, respectively, and pulp sharing the same FICA value as calyx. These results were inconsistent with the other antioxidant assays. The slightly higher chelation ability in Fuyu than Jiro may reflect cultivar‐specific differences in acid metabolism or phenolic acid composition, despite both being non‐astringent. Jiro may retain more free acids or unbound flavonol glycosides in both rind and edible parts [32]. It is also related to differences in functional groups involved in iron chelation across the two persimmon cultivars, as siderophores with three bidentate ligands are more effective in binding metal ions, suggesting more catecholate or hydroxamate chelators in Fuyu's calyx than Jiro [32]. However, since the assay can respond not only to phenolic compounds but also to peptides and sulfates, the results may be overestimated.

#### 3.2.3 Non‐Radical Redox‐Potential (FRAP, RPA, and TAC)

By evaluating the sample's capacity to reduce the Fe^3^^+^ TPTZ complex to its blue‐colored Fe^2^^+^ TPTZ form, the FRAP assay measures electron‐donating ability [6]. In this study, FRAP again highlighted the strong antioxidant potential of persimmon calyx tissues, with Fuyu and Jiro calyx extracts recording 92.35 and 89.69 mg TE/g DW, respectively. These values were markedly higher than those of all edible tissues, confirming the calyx as a concentrated source of reducing agents. Since the FRAP response is greatly influenced by electron‐rich phenolic structures, these findings indicate the presence of catechol‐ and galloyl‐containing compounds in persimmon calyces.

For edible tissues, the FRAP values were substantially lower and displayed clear cultivar‐ and tissue‐specific differences. In the rind, Jiro showed slightly higher activity than Fuyu (5.32 vs. 4.17 mg TE/g DW). A similar pattern was observed in pulp, where Jiro reached 6.81 mg/g compared with 4.54 mg/g in Fuyu. The most pronounced disparity occurred in the inner pulp: Jiro recorded 3.59 mg/g, nearly 6 times higher than the Fuyu inner pulp value of 0.60 mg/g. As FRAP trends generally mirror free radical scavenging patterns, these results support the ability of persimmon phenolics to reduce ferric ions [33]. They also suggest that Jiro retains more electron‐donating antioxidant compounds in its edible tissues, particularly toward the fruit center. The reduced FRAP activity in Fuyu inner pulp may reflect cultivar‐specific differences in phenolic biosynthesis, tissue permeability, or the degree of phenolic polymerization during maturation [28].

Collectively, these findings indicate that although both cultivars are classified as non‐astringent, they exhibit considerably different distributions of redox‐active antioxidants across fruit tissues. When compared with Li, Du [26], the general trend observed for rind and pulp was similar, although direct comparison with other persimmon tissues was limited due to the scarcity of relevant studies.

In line with the FRAP results, the reducing power assay (RPA) demonstrated a similar pattern of antioxidant activity across tissues, with both calyx samples showing the strongest reducing capacity. Fuyu calyx recorded the highest RPA value at 42.40 mg TE/g, followed closely by Jiro calyx at 46.02 mg TE/g. These results reaffirm the calyx as the primary site of redox potential in both cultivars. The edible tissues exhibited substantially lower RPA values, ranging from 4.19 to 1.46 mg TE/g in Fuyu and 5.77 to 3.59 mg TE/g in Jiro. Notably, Jiro pulp (5.77 mg/g) displayed stronger reducing power than Fuyu pulp (3.36 mg/g), contrasting with the TPC trend where Fuyu generally showed higher phenolic levels. This discrepancy may reflect differences in antioxidant composition, as reducing power is closely linked to overall antioxidant activity [33]. It suggests that Jiro pulp may contain higher levels of non‐phenolic reducing agents, such as ascorbate or amino acids, which contribute to RPA but not to TPC. Additionally, RPA values in inner pulp remained low for both cultivars, supporting the notion that central fruit tissues in non‐astringent persimmons possess limited redox‐active antioxidant content, likely due to reduced vascular flow and restricted metabolite transport during fruit enlargement. The general trend observed here aligns with findings from Jang, Jo [15], who also reported substantially higher reducing power in calyx extracts compared with peel and flesh, although direct comparison is limited because their values were expressed as IC_50_.

The TAC assay, also known as the phosphor‐molybdenum assay, integrates both electron‐donating and hydrogen‐atom‐transfer mechanisms via Mo^6^^+^ reduction and provides an estimate of total antioxidant capacity. In this study, the superior antioxidant properties of the calyx were again evident. Fuyu calyx displayed the highest TAC value at 28.39 mg TE/g DW, followed closely by Jiro calyx at 27.25 mg TE/g DW. These values were consistent with the trends observed for FRAP and RPA, reinforcing the role of the calyx as the most antioxidant‐rich tissue among the four parts analyzed. TAC values for rind were moderate, with Fuyu (3.37 mg TE/g DW) showing slightly higher activity than Jiro (3.11 mg TE/g DW). Among edible tissues, TAC was lowest in inner pulp, with Jiro showing higher values (pulp: 5.16 mg TE/g DW; inner pulp: 2.99 mg TE/g DW) than Fuyu (4.52 mg TE/g DW; inner pulp: 2.53 mg TE/g DW) across all edible fractions. Since TAC is often correlated with total phenolic content, the patterns observed generally mirrored TPC trends. The agreement between TAC and other redox‐based assays further supports the reliability of the observed antioxidant patterns and highlights the influence of tissue type and cultivar on antioxidant capacity in persimmons. Due to the limited number of studies applying the TAC assay to persimmon fruit, direct comparison with previous research was not possible.

#### 3.2.4 Correlation Between Phenolic Content and Antioxidant Assays

The Pearson correlation analysis demonstrated a strong and consistent relationship between TPC and most antioxidant assays (Table 2). TPC showed highly significant positive correlations with DPPH (*r* = 0.994), FRAP (*r* = 0.988), ABTS^+^ (*r* = 0.991), RPA (*r* = 0.992), ·OH‐RSA (*r* = 0.985), and TAC (*r* = 0.987), indicating that phenolics were the primary contributors to both radical‐scavenging and redox‐based antioxidant activity. Total flavonoid content (TFC) also correlated well with these assays (*r* = 0.917–0.935), reinforcing the functional importance of flavonoid‐rich tissues.

**TABLE 2:** Pearson's correlation matrix among phenolic‐related parameters (TPC, TFC, and TTC) and antioxidant assays (DPPH, FRAP, ABTS+, ·OH‐RSA, FICA, and TAC) in persimmon samples.

	TPC	TFC	DPPH	FRAP	ABTS^+^	RPA	·OH‐RSA	FICA
TFC	0.91^a^							
TCT	—	—						
DPPH	0.994^a^	0.935^a^						
FRAP	0.988^a^	0.934^a^	0.999^a^					
ABTS^+^	0.991^a^	0.927^a^	0.997^a^	0.995^a^				
RPA	0.992^a^	0.931^a^	0.999^a^	0.999^a^	0.996^a^			
^·^OH‐RSA	0.985^a^	0.936^a^	0.996^a^	0.996^a^	0.991^a^	0.994^a^		
FICA	−0.666	−0.543	−0.669	−0.682	−0.677	−0.692	−0.62	
TAC	0.987^a^	0.917^a^	0.996^a^	0.999^a^	0.992^a^	0.998^a^	0.994^a^	−0.697

^a^ Significant correlation with *p* < 0.05.

Abbreviations: TPC, total phenolic content; TFC, total flavonoid content; DPPH, 2,2‐diphenyl‐1‐picrylhydrazyl radical scavenging activity; FRAP, ferric reducing antioxidant power; ABTS+, 2,2'‐azino‐bis(3‐ethylbenzothiazoline‐6‐sulfonic acid) radical scavenging activity; RPA, reducing power activity; ·OH, hydroxyl radical scavenging activity; FICA, ferric ions chelating activity; TAC, total antioxidant capacity.

In contrast, FICA showed poor correlations with TPC (*r* = −0.666), TFC (*r* = −0.543), and all antioxidant indices. This inverse trend reflects a key limitation of the FICA method in this matrix. The assay is non‐specific and can respond not only to phenolics but also to peptides and sulfur‐containing compounds present in plant extracts, leading to over‐ or underestimation of true polyphenol‐derived chelating activity [34]. Moreover, because metal chelation is structurally dependent, certain phenolics may coordinate iron ineffectively under assay conditions, yielding poor agreement with redox‐based and total bioactive assays that evaluate distinct mechanisms unrelated to electron transfer or hydrogen donation [34]. Therefore, the negative correlations should not be viewed as contradictory, but as evidence of assay‐specific constraints and structural selectivity inherent to metal‐chelation‐based antioxidant estimation. Collectively, these limitations suggest that FICA may be unsuitable as a primary indicator of antioxidant capacity in persimmon tissues.

### 3.3 Phenolic Profiling by LC‐ESI‐QTOF‐MS/MS

This study employed LC‑ESI‑QTOF‑MS/MS, operated in both negative and positive ionization modes, to achieve a comprehensive qualitative characterization of phenolic compounds across the eight persimmon samples. Phenolics were identified based on precursor ion *m/z* values together with diagnostic MS/MS fragmentation patterns. The analysis revealed a wide diversity of phenolic constituents distributed among the different tissues of both Fuyu and Jiro cultivars. In total, the identified compounds were grouped into five major categories: phenolic acids (31), flavonoids (45), lignans (4), stilbenes (2), and other polyphenols (12), as summarized in Table 3. Representative LC‐ESI‐QTOF‐MS/MS base peak chromatograms and MS/MS spectra are provided in Figures S1 and S2 to support compound identification.

**TABLE 3:** Comprehensive characterization of phenolic compounds in different persimmon cultivars and parts by LC‐ESI‐QTOF‐MS/MS.

Sr no.		Proposed compounds	Molecular formula	RT (min)	Ionization (ESI+/ESI‐)	Molecular weight	Theoretical (*m/z*)	Observed (*m/z*)	Error (ppm)	MS^2^ product ions	Persimmon samples
Phenolic acids											
Hydroxybenzoic acids											
1		Ellagic acid glucoside	C_20_H_16_O_13_	32.599	[M‐H]^−^	464.0597	463.0524	463.0544	4.32	301	MJC
2		Ellagic acid arabinoside	C_19_H_14_O_12_	48.032	[M‐H]^−^	434.0518	433.0445	433.0434	−2.54	300	CFC
3		Benzoic acid	C_7_H_6_O_2_	51.344	[M‐H]^−^	122.0356	121.0283	121.0283	0.00	77	^a^MJR, MJV, CFC, CFP, and CFV
4		2‐Hydroxybenzoic acid	C_7_H_6_O_3_	54.015	^b^[M‐H]^−^	138.0307	137.0234	137.0234	0.00	93	^a^CFR, MJP, CFC, CFP, CFV, and MJC
5		Protocatechuic acid	C_7_H_6_O_4_	54.633	[M‐H]^−^	154.0256	153.0183	153.0188	3.27	109	^a^CFP and CFV
6		Gallic acid 4‐*O*‐glucoside	C_13_H_16_O_10_	55.477	[M‐H]^−^	332.0728	331.0655	331.066	1.51	169, 125	^a^CFR and MJC
7		Ellagic acid	C_14_H_6_O_8_	55.525	^b^[M‐H]^−^	302.0035	300.9962	300.9961	−0.33	300	^a^CFR, MJV, CFP, and MJC
8		Gallic acid 3‐*O*‐gallate	C_14_H_10_O_9_	55.548	[M‐H]^−^	322.0316	321.0243	321.0247	1.25	169	^a^MJC
9		Ellagic acid acetyl‐arabinoside	C_21_H_16_O_13_	56.907	[M‐H]^−^	476.0572	475.0499	475.0518	4.00	301	^a^MJP, MJC, and MJR
Hydroxycinnamic acids											
10		Rosmarinic acid	C_18_H_16_O_8_	4.043	[M‐H]^−^	360.0833	359.076	359.0777	4.73	179	CFV
11		Caffeic acid 3‐sulfate	C_9_H_8_O_7_S	34.773	[M‐H]^−^	259.9978	258.9905	258.9908	1.16	179, 135	MJP
12		Caffeic acid	C_9_H_8_O_4_	45.712	^b^[M‐H]^−^	180.041	179.0337	179.0342	2.79	143, 133	^a^MJR, and MJC
13		Verbascoside	C_29_H_36_O_15_	45.878	[M‐H]^−^	624.1996	623.1923	623.1923	0.00	477, 461, 315, 135	CFP
14		*p*‐Coumaroyl tyrosine	C_18_H_17_NO_5_	53.62	[M‐H]^−^	327.1087	326.1014	326.1016	0.61	282	^a^MJR, CFP, and CFV
15		1‐Sinapoyl‐2‐feruloylgentiobiose	C_33_H_40_O_18_	54.14	[M‐H]^−^	724.2223	723.215	723.218	4.15	529, 499	MJV
16		Chicoric acid	C_22_H_18_O_12_	54.615	[M‐H]^−^	474.0793	473.072	473.0731	2.33	293, 311	^a^CFV, CFP
17		1,2,2'‐Triferuloylgentiobiose	C_42_H_46_O_20_	54.693	[M‐H]^−^	870.2586	869.2513	869.2481	−3.68	693, 517	MJP
18		Cinnamic acid	C_9_H_8_O_2_	55.14	^b^[M‐H]^−^	148.0515	147.0442	147.0444	1.36	103	^a^CFR and MJC
19		3‐Caffeoylquinic acid	C_16_H_18_O_9_	55.196	^b^[M‐H]^−^	354.0932	353.0859	353.0862	0.85	253, 190, 144	^a^MJV, MJP, CFP, and MJC
20		Caffeoyl glucose	C_15_H_18_O_9_	55.238	[M‐H]^−^	342.0934	341.0861	341.0874	3.81	179, 161	MJV
21		4,5‐Dicaffeoylquinic acid	C_25_H_24_O_12_	55.399	^b^[M‐H]^−^	516.1255	515.1182	515.1206	4.66	353, 335, 191, 179	^a^CFV and MJC
22		Caffeoyl C1‐glucuronide	C_15_H_16_O_10_	55.426	[M‐H]^−^	356.0738	355.0665	355.0672	1.97	179	^a^MJC and CFV
23		*p*‐Coumaric acid 4‐*O*‐glucoside	C_15_H_18_O_8_	56.011	[M‐H]^−^	326.1008	325.0935	325.0938	0.92	163	^a^CFV and CFR
24		3‐Sinapoylquinic acid	C_18_H_22_O_10_	56.685	[M‐H]^−^	398.1202	397.1129	397.1118	−2.77	233, 179	^a^CFC, CFV
25		Ferulic acid 4‐*O*‐glucuronide	C_16_H_18_O_10_	57.106	^b^[M‐H]^−^	370.0885	369.0812	369.0813	0.27	193	^a^CFC, MJR, and MJC
26		*p*‐Coumaroyl malic acid	C_13_H_12_O_7_	57.229	[M‐H]^−^	280.058	279.0507	279.0505	−0.72	163, 119	CFC
27		Hydroxycaffeic acid	C_9_H_8_O_5_	57.441	[M‐H]^−^	196.0371	195.0298	195.0296	−1.03	151	CFP
28		5‐Feruloylquinic acid	C_17_H_20_O_9_	57.441	[M‐H]^−^	368.1111	367.1038	367.1028	−2.72	298, 288, 192, 191	^a^CFP, MJR, MJP, and MJV
29		*p*‐Coumaroyl tartaric acid	C_13_H_12_O_8_	57.448	[M‐H]^−^	296.0522	295.0449	295.0442	−2.37	115	MJP
Hydroxyphenylpentanoic acids											
30		5‐(3'‐Methoxy‐4'‐hydroxyphenyl)‐*γ*‐valerolactone	C_12_H_14_O_4_	41.891	^b^[M‐H]^+^	222.0877	223.095	223.0946	−1.79	205	^a^MJC, CFC, CFR, and CFP
Hydroxyphenylpropanoic acids											
31		Dihydrocaffeic acid 3‐*O*‐glucuronide	C_15_H_18_O_10_	55.415	[M‐H]^−^	358.0892	357.0819	357.0821	0.56	181	^a^CFP and CFC
Flavonoids											
Flavanols											
32	Theaflavin 3,3'‐*O*‐digallate		C_43_H_32_O_20_	7.369	^b^[M‐H]^−^	868.1485	867.1412	867.1425	1.50	545, 563, 715	^a^MJP, CFR, and CFV
33	(+)‐Gallocatechin		C_15_H_14_O_7_	19.145	^b^[M‐H]^−^	306.0711	305.0638	305.0638	0.00	261, 219	^a^CFC, CFV, and MJC
34	(‐)‐Epicatechin		C_15_H_14_O_6_	24.115	^b^[M‐H]^−^	290.0764	289.0691	289.069	−0.35	245, 205, 179	^a^CFC, MJR, MJP, CFV, and MJC
35	4'‐*O*‐Methylepigallocatechin		C_16_H_16_O_7_	34.967	[M‐H]^+^	320.0879	321.0952	321.0948	−1.25	302	MJC
36	Cinnamtannin A2		C_60_H_50_O_24_	51.81	[M‐H]^−^	1154.2726	1153.2653	1153.2646	0.61	739	CFC
37	4''‐*O*‐Methylepigallocatechin 3‐*O*‐gallate		C_23_H_20_O_11_	53.976	[M‐H]^−^	472.1	471.0927	471.0934	1.49	169, 319	CFC
38	Procyanidin trimer C1		C_45_H_38_O_18_	54.312	[M‐H]^−^	866.2054	865.1981	865.2016	4.05	739, 713, 695	^a^CFP and MJR
39	Procyanidin dimer B7		C_30_H_26_O_12_	57.866	^b^[M‐H]^−^	578.142	577.1347	577.1359	2.08	451	^a^CFV, MJR, CFV, and MJC
Flavanones											
40		Sakuranetin	C_16_H_14_O_5_	33.989	^b^[M‐H]^+^	286.0825	287.0898	287.0897	−0.35	269, 203, 201, 175	^a^MJC and CFR
41		Hesperetin 3'‐*O*‐glucuronide	C_22_H_22_O_12_	54.152	[M‐H]^−^	478.1125	477.1052	477.1058	1.26	301, 175, 113, 85	CFV
42		Isoxanthohumol	C_21_H_22_O_5_	54.609	[M‐H]^−^	354.148	353.1407	353.1405	−0.57	338, 309	^a^MJP, CFC, and CFV
43		Hesperetin 5,7‐*O*‐diglucuronide	C_28_H_30_O_18_	54.961	[M‐H]^−^	654.1424	653.1351	653.1367	2.45	477, 301, 286, 242	MJC
44		Hesperetin 3'‐sulfate	C_16_H_14_O_9_S	55.03	[M‐H]^−^	382.0351	381.0278	381.0294	4.20	301, 286, 257, 242	MJV
45		Naringin 4'‐*O*‐glucoside	C_33_H_42_O_19_	55.04	[M‐H]^−^	742.2352	741.2279	741.2274	−0.67	433, 271	CFV
46		Narirutin	C_27_H_32_O_14_	56.455	[M‐H]^−^	580.1774	579.1701	579.1702	0.17	271	CFP
47		Eriocitrin	C_27_H_32_O_15_	57.45	[M‐H]^−^	596.1735	595.1662	595.1657	−0.84	431, 287	^a^CFC and MJR
Flavones											
48		Scutellarein	C_15_H_10_O_6_	35.153	^b^[M‐H]^+^	286.0471	287.0544	287.0544	0.00	269, 259	^a^MJC, MJP, and CFP
49		Apigenin 6‐C‐glucoside	C_21_H_20_O_10_	48.479	[M‐H]^−^	432.1025	431.0952	431.0963	2.55	413, 341, 311	^a^CFR, MJR, and CFV
50		Apigenin 6,8‐di‐C‐glucoside	C_27_H_30_O_15_	53.691	[M‐H]^−^	594.1584	593.1511	593.152	1.52	503, 473	^a^MJV and CFV
51		Isorhoifolin	C_27_H_30_O_14_	54.082	[M‐H]^−^	578.1621	577.1548	577.1576	4.85	413, 269	^a^CFR and CFP
52		Apigenin 7‐*O*‐(6''‐malonyl‐apiosyl‐glucoside)	C_29_H_30_O_17_	54.483	[M‐H]^−^	650.1468	649.1395	649.1424	4.47	605	CFV
53		Apigenin 7‐*O*‐diglucuronide	C_27_H_26_O_17_	55.486	[M‐H]^−^	622.1193	621.112	621.1122	0.32	269	^a^CFV, MJC, and MJV
54		Nobiletin	C_21_H_22_O_8_	57.845	[M‐H]^+^	402.1314	403.1387	403.1383	−0.99	388, 373, 355, 327	MJC
Flavonols											
55		Myricetin 3‐*O*‐rhamnoside	C_21_H_20_O_12_	32.804	[M‐H]^−^	464.0909	463.0836	463.0834	−0.43	317	^a^CFC and CFR
56		Quercetin 3‐*O*‐rhamnoside	C_21_H_20_O_11_	35.113	[M‐H]^−^	448.0962	447.0889	447.0889	0.00	301	^a^CFC, MJV, and CFR
57		Kaempferol 3‐*O*‐(2''‐rhamnosyl‐galactoside) 7‐*O*‐rhamnoside	C_33_H_40_O_19_	53.709	[M‐H]^−^	740.2173	739.21	739.2128	3.79	593, 447, 285	MJC
58		Kaempferol 3‐*O*‐glucosyl‐rhamnosyl‐galactoside	C_33_H_40_O_20_	54.287	[M‐H]^−^	756.208	755.2007	755.2006	−0.13	285	^a^MJP, MJR, MJV, CFC, CFR, and CFV
59		Quercetin 3‐*O*‐glucosyl‐xyloside	C_26_H_28_O_16_	54.733	[M‐H]^−^	596.137	595.1297	595.1306	1.51	265, 138, 116	CFR
60		Quercetin 3'‐sulfate	C_15_H_10_O_10_S	54.963	[M‐H]^−^	381.9995	380.9922	380.9914	−2.10	301	MJP
61		Isorhamnetin 4'‐*O*‐glucuronide	C_22_H_20_O_13_	55.262	[M‐H]^−^	492.0873	491.08	491.0812	2.44	315, 300, 272, 255	MJP
62		Patuletin 3‐*O*‐glucosyl‐(1‐>6)‐[apiosyl(1‐>2)]‐glucoside	C_33_H_40_O_22_	55.549	^b^[M‐H]^−^	788.1992	787.1919	787.1958	4.95	625, 463, 301, 271	^a^CFC and CFP
63		Quercetin 3‐*O*‐rutinoside	C_27_H_30_O_16_	56.094	[M‐H]^−^	610.1528	609.1455	609.144	−2.46	447, 285	MJP
64		Quercetin 3'‐*O*‐glucuronide	C_21_H_18_O_13_	57.326	[M‐H]^−^	478.0758	477.0685	477.0691	1.26	301	MJP
65		Spinacetin 3‐*O*‐(2	C_43_H_48_O_24_	57.668	[M‐H]^−^	948.2539	947.2466	947.2476	1.06	741, 609, 301	MJV
66		Myricetin 3‐*O*‐rutinoside	C_27_H_30_O_17_	57.772	[M‐H]^−^	626.1491	625.1418	625.1425	1.12	301	^a^CFC, MJC, CFP, and CFV
Isoflavonoids											
67		Formononetin 7‐*O*‐glucuronide	C_22_H_20_O_10_	4.044	[M‐H]^−^	444.1056	443.0983	443.0982	−0.23	267, 252	^a^MJP and MJC
68		Violanone	C_17_H_16_O_6_	4.06	^b^[M‐H]^−^	316.0925	315.0852	315.0867	4.76	300, 285, 135	^a^CFP and MJC
69		5,6,7,3',4'‐Pentahydroxyisoflavone	C_15_H_10_O_7_	32.521	^b^[M‐H]^+^	302.0411	303.0484	303.0491	2.31	285, 257	^a^MJC, MJP, and CFC
70		Sativanone	C_17_H_16_O_5_	53.881	[M‐H]^−^	300.0986	299.0913	299.0922	3.01	284, 269, 225	MJR
71		2‐Dehydro‐*O*‐desmethylangolensin	C_15_H_12_O_4_	54.185	[M‐H]^−^	256.0716	255.0643	255.0651	3.14	135, 119	MJV
72		3'‐*O*‐Methylviolanone	C_18_H_18_O_6_	54.565	[M‐H]^−^	330.1093	329.102	329.1029	2.73		MJR
73		6''‐*O*‐Acetyldaidzin	C_23_H_22_O_10_	57.448	[M‐H]^−^	458.1222	457.1149	457.1129	−4.38	221	CFR
Dihydroflavonols											
74		Dihydroquercetin 3‐*O*‐rhamnoside	C_21_H_22_O_11_	54.464	^b^[M‐H]^−^	450.1158	449.1085	449.1087	0.45	256, 284, 299, 314	^a^CFP and MJC
Dihydrochalcones											
75		3‐Hydroxyphloretin 2'‐*O*‐glucoside	C_21_H_24_O_11_	55.688	[M‐H]^−^	452.1318	451.1245	451.1246	0.22	289, 273	^a^CFR and CFC
76		3‐Hydroxyphloretin 2'‐*O*‐xylosyl‐glucoside	C_26_H_32_O_15_	57.668	[M‐H]^−^	584.1728	583.1655	583.1677	3.77	289	MJV
Lignans											
77		Schisandrin C	C_22_H_24_O_6_	46.462	[M‐H]^+^	384.1561	385.1634	385.1631	−0.78	370, 315, 300	MJC
78		Pinoresinol	C_20_H_22_O_6_	55.12	[M‐H]^−^	358.14	357.1327	357.1337	2.80	342, 327, 313, 221	CFR
79		7‐Oxomatairesinol	C_20_H_20_O_7_	55.219	^b^[M‐H]^+^	372.1187	373.126	373.1259	−0.27	358, 343, 328, 325	^a^MJC, CFP, and CFV
80		Schisanhenol	C_23_H_30_O_6_	57.737	[M‐H]^+^	402.2044	403.2117	403.2129	2.98	331, 354, 385	MJC
Stilbenes											
81		Pterostilbene	C_16_H_16_O_3_	54.98	[M‐H]^−^	256.1081	255.1008	255.0996	−4.70	227, 241	CFV
82		*trans*‐Resveratrol	C_14_H_12_O_3_	55.268	[M‐H]^−^	228.0765	227.0692	227.07	3.52	212, 185, 157, 143	CFV
Other polyphenols											
Hydroxybenzaldehydes											
83		*p*‐Anisaldehyde	C_8_H_8_O_2_	50.68	^b^[M‐H]^+^	136.0517	137.059	137.0585	−3.65	122, 109	^a^MJC and CFV
84		Vanillin	C_8_H_8_O_3_	53.837	^b^[M‐H]^−^	152.0464	151.0391	151.0389	−1.32	136, 92	^a^MJC and CFV
Hydroxybenzoketones											
85		2,3‐Dihydroxy‐1‐guaiacylpropanone	C_10_H_12_O_5_	54.613	[M‐H]^−^	212.0674	211.0601	211.0597	−1.90	167, 123, 105, 93	CFP
Hydroxycoumarins											
86		Scopoletin	C_10_H_8_O_4_	55.016	^b^[M‐H]^−^	192.0408	191.0335	191.0342	3.66	176	^a^CFR, CFP, and MJC
Hydroxyphenylpropenes											
87		Eugenol	C_10_H_12_O_2_	53.588	[M‐H]^+^	164.0831	165.0904	165.0905	0.61	149, 137, 133, 124	MJC
Other polyphenols											
88		Salvianolic acid B	C_36_H_30_O_16_	4.039	[M‐H]^−^	718.1554	717.1481	717.1512	4.32	519, 339, 321, 295	MJC
89		Arbutin	C_12_H_16_O_7_	53.948	^b^[M‐H]^−^	272.0882	271.0809	271.0809	0.00	109	^a^MJV, MJR, CFR, and MJC
Phenolic terpenes											
90		Epirosmanol	C_20_H_26_O_5_	54.014	^b^[M‐H]^+^	346.1777	347.185	347.1849	−0.29	301, 241, 231	^a^MJC, MJP, CFR, CFP, and CFV
Tyrosols											
91		3,4‐DHPEA‐EDA	C_17_H_20_O_6_	4.043	[M‐H]^−^	320.1228	319.1155	319.117	4.70	275, 195	CFV
92		Demethyloleuropein	C_24_H_30_O_13_	55.218	[M‐H]^−^	526.1702	525.1629	525.1614	−2.86	301, 241, 231	^a^MJR, MJV, and CFC
93		3,4‐DHPEA‐AC	C_10_H_12_O_4_	55.227	^b^[M‐H]^−^	196.0718	195.0645	195.0648	1.54	135	^a^CFP and MJC
94		Hydroxytyrosol 4‐*O*‐glucoside	C_14_H_20_O_8_	55.89	^b^[M‐H]^−^	316.1136	315.1063	315.1063	0.00	153, 123	^a^CFV, CFR, CFP, and MJC

^a^ Indicates the compound was detected more than one sample.

^b^ Indicated the compound was detected in both negative mode and positive ionization mode.

Abbreviations: CFC, Fuyu calyx; CFR, Fuyu rind; CFP, Fuyu pulp; CFV, Fuyu inner pulp; MJC, Jiro calyx; MJR, Jiro rind; MJP, Jiro pulp; MJV, Jiro inner pulp.

#### 3.3.1 Phenolic Acids

In this research, a total of 31 phenolic acids were annotated, comprising hydroxybenzoic acids (9), hydroxycinnamic acids (20), hydroxyphenylpentanoic acids (1), and hydroxyphenylpropanoic acids (1). Hydroxybenzoic acids such as gallic acid 4‐*O*‐glucoside and gallic acid 3*‐O*‐gallate were detected in Jiro calyx (MJC) and Fuyu rind (CFR) extract samples. Compound **6** was identified as gallic acid 4‐*O*‐glucoside based on a deprotonated molecular ion at *m/z* 331 and confirmed via fragment ions at *m/z* 169 and 125, indicate the loss of a glucoside unit (162 Da) along with CO_2_ (44 Da), consistent with Subbiah, Zhong [35]. Compound **7** (Ellagic acid) tentatively identified at *m/z* 301 at RT 55 min in [M‐H]^−^, resulting product ions *m/z* 300 and 257 with characteristic loss of lactone moieties (44 Da) and hydrogen radical (1 Da). Results supported by previous studies reported ellagic acid [C_14_H_8_O_6_] is identified in fruit samples (Renai et al. 2024) [36] recognized as one of the top contributors of antioxidant activities resulting from phenolic O─H bond cleavage free H‐radicals (Cai et al. 2025) [37].

Compound **4** was identified as 2‐hydroxybenzoic acid at *m/z* 137 in negative ionization mode, present across pulp, peel, and calyx samples, supporting the variable ·OH scavenging values in these tissues (Martínez‐Las Heras, Quifer‐Rada [38]. Gallic acid 3‐*O*‐gallate (Compound **8**) in [M − H]^−^ mode observed at *m/z* 321 with a characteristic fragment loss at *m/z* 169, resulting from C‐ring cleavage (Taamalli, Iswaldi [39]. These structures, enriched in catechol or trihydroxybenzene moieties, are known for strong antioxidant potential through electron donation and metal chelation [31], which aligns with the elevated antioxidant activities observed in calyx and rind tissues.

Hydroxycinnamic acids were the most identified phenolic acids, including caffeic acid and its conjugates, broadly distributed across edible and non‐edible tissues. Compounds 11 and 12 were assigned as caffeic acid and its 3‐sulfate derivative, tentatively identified in [M − H]^−^ ions with observed mass [*m/z* 179 and *m/z* 259], respectively, findings were consistent with Schütz, Kammerer [40]. Caffeic acid fragments ions with *m/z* 143 and 133, may resulted due to loss of two water molecules (36 Da) and H‐COOH (46 Da), respectively. Caffeic acid 3‐sulfate produced fragments at *m/z* 179 and 135, corresponding to SO_3_ and CO_2_, losses confirmed via fragmentation patterns reported by Zhong, Robinson [41]. Conjugated derivatives including caffeoyl glucose, caffeoyl C1‐glucuronide, and 3‐caffeoylquinic acid were identified in most pulp samples at *m/z* 341, 355, and 353, respectively, confirming the presence of bioavailable hydroxycinnamates in edible tissues.

Compound 30, identified as 5‐(3′‐methoxy‐4′‐hydroxyphenyl)‐γ‐valerolactone, was the only hydroxyphenylpentanoic acid detected. It appeared in both calyx samples and most fuyu tissues at *m/z* 223 in positive mode of ionization with fragment ion of *m/z* 205, consistent with the pattern reported by Shi, Sejpal [42]. The sole hydroxyphenylpropanoic acid observed was dihydrocaffeic acid 3‐*O*‐glucuronide, detected at *m/z* 357 in negative ionization mode in fuyu calyx and pulp samples. Its fragmentation yielded an *m/z* 181 ion, corresponding to a glucuronic acid loss (Calani, Ounnas [43].

#### 3.3.2 Flavonoids

A total of 45 flavonoids were identified, making them the most identified and structurally diverse phenolic group detected across all persimmon samples. Among flavonoid compounds: flavanols (8), flavanones (8), flavones (7), flavonols (12), isoflavonoids (7), dihydroflavonols (1), and dihydrochalcones (2) were tentatively detected.

Among the flavonoids identified, (+)‐gallocatechin and (–)‐epicatechin were structurally confirmed and were detected in calyx tissues, also present in several edible tissues. Compound 33 was identified as (+)‐gallocatechin in negative ionization mode, characterized by a deprotonated molecular ion [M–H]^−^ at *m/z* 305 and fragment ions at *m/z* 261 and 219. The product ion observed with *m/z* 261 fragment corresponds to CO_2_ loss (Martínez‐Las Heras, Quifer‐Rada [38], while other fragment ions observed are *m/z* 221 and *m/z* 219 reflects the possible slight difference of mass due to minor variation in fragmentation.

Compound 34 [epicatechin] was also detected in negative mode, with product ion *m/z* 289 resulted from the characteristic fragment loss with observed *m/z* 245, 205, and 179. The co‐presence of (+)‐gallocatechin and (–)‐epicatechin in calyx tissues provides a mechanistic explanation for their high antioxidant activity, as both compounds possess strong electron‐donating capacity and efficient redox behavior [44]. Their abundance in calyx tissues aligns with the elevated FRAP, RPA, and TAC values observed, and highlights their potential relevance in antioxidant‐focused applications [44].

Other important flavonols including; myricetin 3‐*O*‐rhamnoside [*m/z* 463, RT 32 min], Quercetin 3‐*O*‐rhamnoside [*m/z* 447, RT 35 min] and Kaempferol 3‐*O‐*glucosyl‐rhamnosyl‐galactoside [*m/z* 755, RT 54 min] were observed in negative mode of ionization in at in various samples of fuyu calyx, rind, pulp and jiro rind and pulp samples.

#### 3.3.3 Lignan and Stilbenes

Four (4) lignan compounds were detected across the persimmon fruit tissues, with notable compounds including Schisandrin C and Schisanhenol. Compound 77 (Schisandrin C) and compound 80 (Schisanhenol) were detected in positive mode of ion mode and were detected in Jiro calyx. Schisandrin C showed an [M + H]^+^ ion at *m/z* 385 with fragment ions at *m/z* 370, 315, and 300, while Schisanhenol displayed an [M + H]^+^ ion at *m/z* 403 and produced fragments at *m/z* 331, 354, and 385.

In addition, two stilbenes were exclusively found in Fuyu inner pulp. Compound 81 was identified as pterostilbene, detected in negative ion mode with an [M–H]^−^ ion at *m/z* 255 and fragment ions at *m/z* 227 and 241. The major ion showed a slight deviation from the *m/z* 256 reported by Raji, Amad [45], which may be resulted due to minor methodological or instrument variations. Compound 82 was observed as *trans*‐resveratrol, showing an [M–H]^−^ ion at *m/z* 227 with characteristic fragments at *m/z* 212, 185, 157, and 143, supporting its structural identification.

#### 3.3.4 Other Polyphenols

A total of 12 additional polyphenols were identified in this study, including hydroxybenzaldehydes (2), hydroxybenzoketones (1), hydroxycoumarins (1), hydroxyphenylpropenes (1), phenolic terpenes (1), tyrosols (4), salvianolic acid B, and arbutin.

Among these compounds, vanillin (compound 84) was identified in the [M–H]^−^ ion mode at *m/z* 151, with dominant fragment ions at *m/z* 136 and 92. These correspond to the sequential loss of CH_3_ and CO_2_, consistent with the fragmentation patterns described by Wu, Salionov [46]. Despite its relatively simple structure, vanillin is known to exhibit moderate antioxidant activity through hydrogen‐atom donation by its phenolic hydroxyl group, or through radical quenching via self‐dimerization [46]. Its presence in persimmon inner pulp suggests that it may contribute to the overall antioxidant potential of this tissue through redox interactions.

### 3.4 Quantification of Target Phenolic Compounds by HPLC‐DAD

In total, fifteen phenolic compounds were targeted and analyzed by HPLC‐DAD. Their retention times, listed from earliest to latest, were as follows: gallic acid (13.12 min), protocatechuic acid (15.30 min), catechin hydrate (16.31 min), 3,4‐dihydroxyhydrocinnamic acid (17.03 min), epicatechin (17.17 min), epigallocatechin gallate (17.33 min), 4‐hydroxybenzoic acid (17.46 min), caffeic acid (17.44 min), syringic acid (17.97 min), naringin (19.31 min), *p*‐coumaric acid (19.88 min), sinapic acid (20.11 min), *trans*‐ferulic acid (20.36 min), 3‐hydroxy‐4‐methoxycinnamic acid (20.67 min), and quercetin (23.63 min). By matching the retention times of sample peaks with those of authentic standards, three phenolic compounds were successfully quantified, as summarized in Table 4.In this research, gallic acid, catechin hydrate, and sinapic acid were observed and quantified in sample extracts. The results showed that Fuyu calyx had the highest total phenolic content, as it contained the greatest concentrations of gallic acid (2.1208 mg/g) and catechin hydrate (3.072 mg/g). Notably, gallic acid was detected only in Fuyu calyx, whereas catechin hydrate was also present in Jiro calyx at a similar level (3.0391 mg/g). This distribution pattern aligns with earlier observations that Fuyu calyx exhibited higher TPC and overall antioxidant potential than Jiro calyx.

**TABLE 4:** Phenolic content quantifications in mg/g of dry matter in different parts of the two persimmon cultivars.

		Phenolic compounds (mg/g DW)		
		Gallic acid	Catechin hydrate	Sinapic acid
Fuyu	Calyx	2.1208	3.702	0.8742
	Rind	—	—	0.8712
	Pulp	—	—	0.8736
	Inner pulp	—	—	0.8799
Jiro	Calyx	—	3.0391	0.8857
	Rind	—	—	0.8655
	Pulp	—	—	0.8712
	Inner pulp	—	—	0.8417

*Note*: Values are expressed as mg/g dry weight (DW). “—” indicates that the compound was not detected under the chromatographic conditions employed.

Sinapic acid was quantified across all tissues, supporting its contribution to the modest antioxidant activity detected throughout the samples. In previous research by Zou, Wu [47], the presence of gallic acid in persimmon purée was confirmed by HPLC, although neither catechin hydrate nor sinapic acid was reported. Instead, catechin derivatives such as epicatechin and gallocatechin were identified, which is consistent with the LC‐MS/MS results observed in the present study.

## 4 Conclusion

This study comprehensively assessed the antioxidant potential and phenolic profiles of four persimmon tissues across two non‐astringent cultivars. Fuyu calyx exhibited the highest TPC (38.98 mg GAE/g DW), supported by high levels of gallic acid and catechin hydrate. LC‐MS/MS analysis identified 95 phenolic compounds across the samples, reflecting a broad phytochemical diversity that contributed to the strong radical‐scavenging activities observed. Antioxidant assays showed strong positive correlations with TPC and TFC, whereas FICA displayed negative correlations and unexpectedly elevated values in inner pulp, highlighting limitations of this assay for persimmon matrices. While HPLC‐DAD confirmed major phenolics in calyx tissues, the restricted standard panel and dilution discrepancies may have led to underestimation of total phenolic concentrations. Additionally, differences in fruit maturity and moisture content between individual samples may have influenced extraction efficiency. Collectively, these factors emphasize the need for more standardized sampling protocols and a broader range of quantified phenolics to enhance the accuracy and comparability of compositional and functional assessments in non‐astringent persimmon tissues.

## Conflicts of Interest

The authors declare no conflicts of interest.

## Supporting information




**Supporting File 1**: cbdv71492‐sup‐0001‐SuppMat.docx.

## Data Availability

The data that support the findings of this study are available on request from the corresponding author. The data are not publicly available due to privacy or ethical restrictions.

## References

[1] S. Jain , G. Ranganna , S. Mohapatra , et al., “A Comprehensive Review on Persimmon (Diospyros kaki):Botanical, Horticultural, and Varietal Perspectives,” International Journal of Environment and Climate Change 13, no. 11 (2023): 4437–4457, 10.9734/IJECC/2023/v13i113624.

[2] S. Yaqub , U. Farooq , A. Shafi , et al., “Chemistry and Functionality of Bioactive Compounds Present in Persimmon,” Journal of Chemistry 2016, no. 1 (2016): 3424025.

[3] H. Kumar , R. Dhalaria , S. Guleria , et al., “Non‐Edible Fruit Seeds: Nutritional Profile, Clinical Aspects, and Enrichment in Functional Foods and Feeds,” Critical Reviews in Food Science and Nutrition 64, no. 33 (2024): 13298–13317, 10.1080/10408398.2023.2264973. 37811640

[4] P. Novillo , C. Besada , L. Tian , A. Bermejo , and A. Salvador , “Nutritional Composition of Ten Persimmon Cultivars in the “Ready‐to‐Eat Crisp” Stage. Effect of Deastringency Treatment,” Food and Nutrition Sciences 6, no. 14 (2015): 1296–1306, 10.4236/fns.2015.614135.

[5] M.‐A. Fernandez‐Zamudio , H. Barco , and F. Schneider , “Direct Measurement of Mass and Economic Harvest and Post‐Harvest Losses in Spanish Persimmon Primary Production,” Agriculture 10, no. 12 (2020): 581, 10.3390/agriculture10120581.

[6] H. A. Suleria , C. J. Barrow , and F. R. Dunshea , “Screening and Characterization of Phenolic Compounds and Their Antioxidant Capacity in Different Fruit Peels,” Foods 9, no. 9 (2020): 1206, 10.3390/foods9091206. 32882848 PMC7556026

[7] A. Siddiqa , A. Khaliq , M. T. Sultan , et al., “Characterization of Polyphenolic Compounds and Antioxidant Activities of Tagetes Flowers With Varying Treatments Using LC ‐ ESI ‐ QToF ‐ MS/MS,” Food Science & Nutrition 13, no. 10 (2025): e71047, 10.1002/fsn3.71047. 41049415 PMC12494492

[8] J. Severo , A. Tiecher , F. C. Chaves , J. A. Silva , and C. V. Rombaldi , “Gene Transcript Accumulation Associated With Physiological and Chemical Changes During Developmental Stages of Strawberry Cv. Camarosa,” Food Chemistry 126, no. 3 (2011): 995–1000, 10.1016/j.foodchem.2010.11.107.

[9] I. C. F. R. Ferreira , P. Baptista , M. Vilas‐Boas , and L. Barros , “Free‐Radical Scavenging Capacity and Reducing Power of Wild Edible Mushrooms From Northeast Portugal: Individual Cap and Stipe Activity,” Food Chemistry 100, no. 4 (2007): 1511–1516, 10.1016/j.foodchem.2005.11.043.

[10] A. Ali , Y. M. Bashmil , J. J. Cottrell , H. A. R. Suleria , and F. R. Dunshea , “LC‐MS/MS‐QTOF Screening and Identification of Phenolic Compounds From Australian Grown Herbs and Their Antioxidant Potential,” Antioxidants 10, no. 11 (2021): 1770, 10.3390/antiox10111770. 34829641 PMC8615083

[11] A. Faller and E. Fialho , “Polyphenol Content and Antioxidant Capacity in Organic and Conventional Plant Foods,” Journal of Food Composition and Analysis 23, no. 6 (2010): 561–568, 10.1016/j.jfca.2010.01.003.

[12] M. Del Bubba , E. Giordani , L. Pippucci , A. Cincinelli , L. Checchini , and P. Galvan , “Changes in Tannins, Ascorbic Acid and Sugar Content in Astringent Persimmons During on‐Tree Growth and Ripening and in Response to Different Postharvest Treatments,” Journal of Food Composition and Analysis 22, no. 7‐8 (2009): 668–677.

[13] S. Sakanaka , Y. Tachibana , and Y. Okada , “Preparation and Antioxidant Properties of Extracts of Japanese Persimmon Leaf Tea (kakinoha‐cha),” Food Chemistry 89, no. 4 (2005): 569–575, 10.1016/j.foodchem.2004.03.013.

[14] E. B. Jang , T.‐T. Ho , and S.‐Y. Park , “Effect of Light Quality and Tissue Origin on Phenolic Compound Accumulation and Antioxidant Activity in Camellia japonica calli,” In Vitro Cellular & Developmental Biology—Plant 56, no. 5 (2020): 567–577, 10.1007/s11627-020-10121-9.

[15] I.‐C. Jang , E.‐K. Jo , M.‐S. Bae , et al., “Antioxidant and Antigenotoxic Activities of Different Parts of Persimmon (Diospyros kaki cv. Fuyu) Fruit,” Journal of Medicinal Plants Research 4, no. 2 (2010): 155–160.

[16] T. Akagi , A. Ikegami , Y. Suzuki , et al., “Expression Balances of Structural Genes in Shikimate and Flavonoid Biosynthesis Cause a Difference in Proanthocyanidin Accumulation in Persimmon (Diospyros kaki Thunb.) Fruit,” Planta 230, no. 5 (2009): 899–915, 10.1007/s00425-009-0991-6. 19669159

[17] J. U.‐H. Choe , H.‐Y. Kim , Y.‐J. Kim , E.‐J. Yeo , and C.‐J. Kim , “Antioxidant Activity and Phenolic Content of Persimmon Peel Extracted With Different Levels of Ethanol,” International Journal of Food Properties 17, no. 8 (2014): 1779–1790, 10.1080/10942912.2012.731460.

[18] E. Katsumi , N. Oshima , N. Kagawa , H. Ohara , and N. Hada , “Changes in the Extracted Amounts and Seasonally Variable Constituents of Diospyros kaki at Different Growth Stages,” Journal of Natural Medicines 75, no. 1 (2021): 105–115, 10.1007/s11418-020-01456-z. 33084985

[19] M. A. Tessmer , C. Besada , I. Hernando , B. Appezzato‐da‐Glória , A. Quiles , and A. Salvador , “Microstructural Changes While Persimmon Fruits Mature and Ripen. Comparison Between Astringent and Non‐Astringent Cultivars,” Postharvest Biology and Technology 120 (2016): 52–60, 10.1016/j.postharvbio.2016.05.014.

[20] H. Gibbins and G. Carpenter , “Alternative Mechanisms of Astringency–What is the Role of Saliva?,” Journal of Texture Studies 44, no. 5 (2013): 364–375, 10.1111/jtxs.12022.

[21] C. M. Renard , A. A. Watrelot , and C. L. Bourvellec , “Interactions Between Polyphenols and Polysaccharides: Mechanisms and Consequences in Food Processing and Digestion,” Trends in Food Science & Technology 60 (2017): 43–51, 10.1016/j.tifs.2016.10.022.

[22] J. L. Vázquez‐Gutiérrez , I. Hernando , and A. Quiles , “Changes in Tannin Solubility and Microstructure of High Hydrostatic Pressure‐Treated Persimmon Cubes During Storage at 4°C,” European Food Research and Technology 237, no. 1 (2013): 9–17, 10.1007/s00217-013-2010-1.

[23] E. Jöbstl , J. O'Connell , J. P. A. Fairclough , and M. P. Williamson , “Molecular Model for Astringency Produced by Polyphenol/Protein Interactions,” Biomacromolecules 5, no. 3 (2004): 942–949. 15132685 10.1021/bm0345110

[24] J. Shi , D. Fang , Y. Sui , et al., “Polyphenol Content, Antioxidant Capacity, and Composition in Different Varieties of Sweet Potato (Ipomoea batatas L.) Leaves During Growth Stages,” Scientia Horticulture 342 (2025): 113925, 10.1016/j.scienta.2024.113925.

[25] C. A. Rice‐Evans , N. J. Miller , and G. Paganga , “Structure‐Antioxidant Activity Relationships of Flavonoids and Phenolic Acids,” Free Radical Biology and Medicine 20, no. 7 (1996): 933–956, 10.1016/0891-5849(95)02227-9. 8743980

[26] P.‐M. Li , G.‐R. Du , and F.‐W. Ma , “Phenolics Concentration and Antioxidant Capacity of Different Fruit Tissues of Astringent versus Non‐Astringent Persimmons,” Scientia Horticulturae 129, no. 4 (2011): 710–714, 10.1016/j.scienta.2011.05.024.

[27] I. R. Ilyasov , V. L. Beloborodov , I. A. Selivanova , and R. P. Terekhov , “ABTS/PP Decolorization Assay of Antioxidant Capacity Reaction Pathways,” International Journal of Molecular Sciences 21, no. 3 (2020): 1131, 10.3390/ijms21031131. 32046308 PMC7037303

[28] F. Pu , X.‐L. Ren , and X.‐P. Zhang , “Phenolic Compounds and Antioxidant Activity in Fruits of Six Diospyros kaki Genotypes,” European Food Research and Technology 237, no. 6 (2013): 923–932, 10.1007/s00217-013-2065-z.

[29] J. Treml and K. Šmejkal , “Flavonoids as Potent Scavengers of Hydroxyl Radicals,” Comprehensive Reviews in Food Science and Food Safety 15, no. 4 (2016): 720–738, 10.1111/1541-4337.12204. 33401843

[30] L. Sun , J. Zhang , X. Lu , L. Zhang , and Y. Zhang , “Evaluation to the Antioxidant Activity of Total Flavonoids Extract From Persimmon (Diospyros kaki L.) Leaves,” Food and Chemical Toxicology 49, no. 10 (2011): 2689–2696, 10.1016/j.fct.2011.07.042. 21802475

[31] L. Mira , M. Tereza Fernandez , M. Santos , R. Rocha , M. Helena Florêncio , and K. R. Jennings , “Interactions of Flavonoids With Iron and Copper Ions: A Mechanism for Their Antioxidant Activity,” Free Radical Research 36, no. 11 (2002): 1199–1208, 10.1080/1071576021000016463. 12592672

[32] J. P. Adjimani and P. Asare , “Antioxidant and Free Radical Scavenging Activity of Iron Chelators,” Toxicology Reports 2 (2015): 721–728, 10.1016/j.toxrep.2015.04.005. 28962407 PMC5598521

[33] J. H. Lee , Y. B. Lee , W. D. Seo , S. T. Kang , J. W. Lim , and K. M. Cho , “Comparative Studies of Antioxidant Activities and Nutritional Constituents of Persimmon Juice (Diospyros kaki L. cv. Gapjubaekmok),” Preventive Nutrition and Food Science 17, no. 2 (2012): 141–151, 10.3746/pnf.2012.17.2.141. 24471076 PMC3866757

[34] N. B. Sadeer , D. Montesano , S. Albrizio , et al., “The Versatility of Antioxidant Assays in Food Science and Safety—Chemistry, Applications, Strengths, and Limitations,” Antioxidants 9, no. 8 (2020): 709, 10.3390/antiox9080709. 32764410 PMC7464350

[35] V. Subbiah , B. Zhong , M. A. Nawaz , C. J. Barrow , F. R. Dunshea , and H. A. R. Suleria , “Screening of Phenolic Compounds in Australian Grown Berries by LC‐ESI‐QTOF‐MS/MS and Determination of Their Antioxidant Potential,” Antioxidants 10, no. 1 (2021): 26, 10.3390/antiox10010026. PMC7824486 33383900

[36] L. Renai , D. Bonetti , G. Bonaccorso , et al., “First Data on the (Poly)phenolic Profiling of Farmacista Honorati Persimmon Fruit (Diospyros kaki Thunb.) at Commercial Harvest and After Treatments for Astringency Removal,” Plants 13, no. 13 (2024): 1768, 10.3390/plants13131768. 38999608 PMC11244366

[37] R. Cai , H. Wang , C. Yu , et al., “Phytochemicals, Antioxidant Activity and Potential Mechanism of the Astragalus Sinicus L. Flower Extracts Against AAPH‐Induced Oxidative Stress in HepG2 Cells,” Future Foods 11 (2025): 100588, 10.1016/j.fufo.2025.100588.

[38] R. M.‐L. Heras , P. Quifer‐Rada , A. Andrés , et al., “III. 3. A Comprehensive Characterization of Persimmon Leaves Polyphenols by High Resolution Mass Spectrometry (LC–ESI‐LTQ‐ORBITRAP‐MS),” Valorización del Cultivo del Caqui 23 (2016): 87.

[39] A. Taamalli , I. Iswaldi , D. Arráez‐Román , A. Segura‐Carretero , A. Fernández‐Gutiérrez , and M. Zarrouk , “UPLC–QTOF/MS for a Rapid Characterisation of Phenolic Compounds From Leaves of Myrtus communis L,” Phytochemical Analysis 25, no. 1 (2014): 89–96, 10.1002/pca.2475. 24115111

[40] K. Schütz , D. R. Kammerer , R. Carle , and A. Schieber , “Characterization of Phenolic Acids and Flavonoids in Dandelion (Taraxacum officinale WEB. ex WIGG.) Root and Herb by High‐Performance Liquid Chromatography/Electrospray Ionization Mass Spectrometry,” Rapid Communications in Mass Spectrometry: An International Journal Devoted to the Rapid Dissemination of Up‐to‐the‐Minute Research in Mass Spectrometry 19, no. 2 (2005): 179–186. 10.1002/rcm.1767 15593267

[41] B. Zhong , N. A. Robinson , R. D. Warner , C. J. Barrow , F. R. Dunshea , and H. A. R. Suleria , “LC‐ESI‐QTOF‐MS/MS Characterization of Seaweed Phenolics and Their Antioxidant Potential,” Marine Drugs 18, no. 6 (2020): 331, 10.3390/md18060331. 32599953 PMC7344666

[42] L. Shi , M. Sejpal , K. Ghafoor , et al., “Ripening Stage and Phenolic Composition Characterization of Fruit From Different Date (Phoenix dactylifera L.) Cultivars in Australia,” Food Science & Nutrition 13, no. 5 (2025): e70221, 10.1002/fsn3.70221. 40321613 PMC12048771

[43] L. Calani , F. Ounnas , P. Salen , et al., “Bioavailability and Metabolism of Hydroxycinnamates in Rats Fed With Durum Wheat Aleurone Fractions,” Food & Function 5, no. 8 (2014): 1738–1746, 10.1039/C4FO00328D. 24977665

[44] M. Grzesik , K. Naparlo , G. Bartosz , and I. Sadowska‐Bartosz , “Antioxidant Properties of Catechins: Comparison With Other Antioxidants,” Food Chemistry 241 (2018): 480–492, 10.1016/j.foodchem.2017.08.117. 28958556

[45] M. Raji , M. A. Amad , and A.‐H. Emwas , “Dehydrodimerization of Pterostilbene During Electrospray Ionization Mass Spectrometry,” Rapid Communications in Mass Spectrometry 27, no. 11 (2013): 1260–1266, 10.1002/rcm.6571. 23650039

[46] X. Wu , D. Salionov , P. Hemberger , F. Vogel , A. Bodi , and S. Bjelić , “Beyond Vanilla: The Dissociation Mechanism of Vanillin in Four Charge States,” Computational and Theoretical Chemistry 1229 (2023): 114340, 10.1016/j.comptc.2023.114340.

[47] B. Zou , J. Wu , Y. Yu , G. Xiao , and Y. Xu , “Evolution of the Antioxidant Capacity and Phenolic Contents of Persimmon During Fermentation,” Food Science and Biotechnology 26, no. 3 (2017): 563–571, 10.1007/s10068-017-0099-x. 30263580 PMC6049598

