# Supplementary material for: Comprehensive Phenolic Profiling and Antioxidant Evaluation of Calyx, Rind, and Edible Tissues From Non‐Astringent Persimmons (Diospyros kaki)
Source: Chem Biodivers. 2026 Jul 27;23(7):e71492. doi: 10.1002/cbdv.71492 (PMC13405436; doi:10.1002/cbdv.71492)
Supplement: Supplementary file 1 — Supporting File 1: cbdv71492‐sup‐0001‐SuppMat.docx. [file CBDV-23-e71492-s001.docx]

**(a)**

**(b)**

**(c)**

**(d)**

**(e)**

**(f)**

**(g)**

**(h)**

**(i)**

**(j)**

**(k)**

**(l)**

**(m)**

**(n)**

**(o)**

**(p)**

**Figure (S1).** LC-ESI-QTOF-MS/MS base peak chromatograms (BPC) for the characterization of phenolic compounds in different tissues of two non-astringent persimmon cultivars. Negative ionization mode: **(a)** CFC (Fuyu calyx), **(b)** CFR (Fuyu rind), **(c)** CFP (Fuyu pulp), **(d)** CFV (Fuyu inner pulp), **(e)** MJC (Jiro calyx), **(f)** MJR (Jiro rind), **(g)** MJP (Jiro pulp), and **(h)** MJV (Jiro inner pulp). Positive ionization mode: **(i)** CFC (Fuyu calyx), **(j)** CFR (Fuyu rind), **(k)** CFP (Fuyu pulp), **(l)** CFV (Fuyu inner pulp), **(m)** MJC (Jiro calyx), **(n)** MJR (Jiro rind), **(o)** MJP (Jiro pulp), and **(p)** MJV (Jiro inner pulp).

**(a)**

**(b)**

**(c)**

**Figure (S2).** Extracted ion chromatogram and mass spectra of (-)-epicatechin (Compound 34, Table 3). **(a)** Extracted ion chromatogram of (-)-epicatechin (C15H14O6) detected at a retention time of 24.115 min in the negative ionization mode (ESI-/[M - H]-) from persimmon tissues. **(b)** Full scan mass spectrum showing the observed precursor ion at m/z 289.0691. **(c)** Product ion spectrum showing characteristic fragment ions at m/z 245, 205, and 179 used for compound identification and characterisation of (-)-epicatechin (Compound 34, Table 3).
